# Exome sequencing identified a novel missense mutation c.464G>A (p.G155D) in Ca^2+^-binding protein 4 (*CABP4*) in a Chinese pedigree with autosomal dominant nocturnal frontal lobe epilepsy

**DOI:** 10.18632/oncotarget.20694

**Published:** 2017-09-05

**Authors:** Zhi-Hong Chen, Chun Wang, Mu-Qing Zhuo, Qiong-Xiang Zhai, Qian Chen, Yu-Xiong Guo, Yu-Xin Zhang, Juan Gui, Zhi-Hong Tang, Xiao-Lu Zeng

**Affiliations:** ^1^ Department of Pediatrics, Guangdong General Hospital, Guangdong Academy of Neuroscience, Guangdong Academy of Medical Sciences, Guangzhou 510080, China

**Keywords:** autosomal dominant nocturnal frontal lobe epilepsy (ADNFLE), Ca^2+^-binding protein 4, Chinese pedigree, whole-exome sequencing

## Abstract

The aim of this study was to identify disease-causing gene mutations in a Chinese family affected with autosomal dominant nocturnal frontal lobe epilepsy (ADNFLE), a 4-generation pedigree of 27 members in the Southern Chinese Han population, including 11 individuals diagnosed with ADNFLE. DNA samples were collected from 15 family members, chinese han people, including seven affected and eight unaffected individuals. None of these patients had night blindness or visual disorders. Four affected individuals were screened for mutations using whole-exome sequencing, and 13 potentially interesting mutations shared by all the four affected individuals were validated using the Sanger sequencing method. Only one novel missense mutation c.464G>A (p.G155D) in the *CABP4* gene, encoding the neuronal Ca^2+^-binding protein 4 (CaBP4), was present in all seven affected individuals in this family as revealed by PCR with blood DNA samples using *CABP4* primers. The mutation was also found in one young unaffected family member, but was absent from 300 unrelated control subjects. The p.G155D mutation, located near the Ca^2+^ binding motif EF-hand 1 and the L-type Ca^2+^ channel (Cav1.4) binding motif within the N-terminal lobe of CaBP4, is predicted to affect protein function according to the bioinformatics tools PolyPhen-2 and SIFT. These findings suggest that mutations in the *CABP4* gene may be linked to ADNFLE.

## INTRODUCTION

Autosomal dominant nocturnal frontal lobe epilepsy (ADNFLE) is an idiopathic focal epilepsy with a spectrum of clinical manifestations. The typical characteristics are clusters of brief motor seizures during light sleep that are often misdiagnosed as nightmares [1, 2, 3]. ADNFLE is a genetically heterogeneous condition, and the roles of genes encoding subunits of neuronal acetylcholine receptors (nAChRs), such as 4, 2, and 2 (CHRNA4, CHRNA2, and CHRNB2), have been clearly established in ADNFLE [4, 5]. Mutations in the *KCNT1* gene, encoding a sodium-gated potassium channel subunit, have been identified in families with severe ADNFLE and accompanying psychiatric features and intellectual disability [6]. However, mutations in these genes only account for a small proportion of families affected with ADNFLE [7, 8], suggesting that other genes contributing to this disorder must exist. In this study, an ADNFLE pedigree in the South Chinese Han population was clinically characterized according to the typical disease phenotype, and whole-exome sequencing was performed in order to identify previously unknown genes linked to ADNFLE.

## RESULTS

### Description of the pedigree

The initial ADNFLE proband was a 37-year-old woman of the Southern Chinese Han population, who presented with brief, clustered, and involuntary motor actions that occurred during sleep. The involuntary motor actions lasted for 0.5 to 1 min and occurred 2 to 8 times per night, manifesting as head scratching, limb flexion, the sensation of being out of breath, tonic stiffening, and vocalization while the patient was conscious. No night blindness, visual disorders, or other abnormal genetic history was noted. The age at onset was 13 years. Examination of the nervous system, physical examination, and cerebral MRI showed no abnormality. The electroencephalogram showed sharp waves and spike and slow waves in bifrontal and bicentral areas during the non-rapid eye movement period. The other affected members of the ADNFLE pedigree showed similar symptoms, but differed in symptom severity (Table 1). All a patients of the pedigree responded well to Topamax and Levetiracetam treatment.

**Table 1:** Main clinical characteristics of seven patients of an ADNFLE pedigree in the Southern Chinese Han population

No.	Sex	Age at time of study (yrs.)	Age at onset (yrs.)	Diurnal seizures	Intellectual disability	Psychiatric disorder	Interictal EEG	MRI	Drug resistance
II4	F	66	8	No	No	No	NA	N	No
III1	F	45	15	Yes	No	No	Rare frontal spikes (sleep recording)	N	No
III3	F	39	11	No	No	No	NA	N	No
III5	F	37	13	No	No	No	bifrontal and bicentral sharp waves and slow–sharp waves (sleep recording)	N	No
III7	F	34	11	No	No	No	bifrontal sharp waves (sleep recording)	N	No
IV1	F	20	12	No	No	No	Right frontotemporal spikes (sleep recording)	N	No
IV6	M	10	6	No	No	No	Rare frontal spikes (sleep recording)	N	No

F, female; M, male; N, normal; NA, not available.

### Exome sequencing

Exome sequencing was performed on the genomic DNA of four affected individuals of the ADNFLE pedigree (Table 1) using NimbleGen Sequence Capture technology (SeqCap EZ library) according to the manufacturer's instructions. The enriched libraries underwent 90 base pair, paired-end sequencing on a HiSeq2000 next-generation sequencing platform (Illumina, San Diego, CA). The sequence data were aligned to the reference human genome (UCSC hg19) using SOAPaligner and variant calling used the SOAPsnp (v.1.03). After filtering PCR duplicates, previously reported variants (dbSNP135 as reported in the UCSC Genome Browser (http://genome.ucsc.edu/), the 1000 Genomes Project (http://www.1000genomes.org) and HapMap 8 (http://hapmap.ncbi.nlm.nih.gov/) databases, with frequency greater than 0.5 %,) and synonymous variants were removed.

Thirteen gene variants were found in the four patients of the ADNFLE pedigree (Table 2), and Sanger sequencing confirmed all the variants. Ten out of thirteen variants were proven to be false-positive. Only three gene mutations (CABP4, FAM72D, MKI67IP) were present in all four patients. However, except for the *CABP4* mutation, the two other gene variants were not only found in affected individuals, but also in other unaffected pedigree members, thus, not separating according to the disease phenotype (Table 3).

**Table 2:** Exome sequencing identified thirteen gene variants in four affected individuals of an ADNFLE pedigree in the Southern Chinese Han population

Chromosome	Gene name	Mutation type	Substitution	Score from SIFT	Prediction from SIFT
Chr11	CABP4	Missense	G155D	0	DaMaging
Chr1	FAM72D	Missense	G82V	0.04	DaMaging
Chr20	FRG1B	Missense	V12A	0.03	DaMaging
Chr20	FRG1B	Missense	D24N	0.03	DaMaging
Chr7	IGF2BP3	Missense	I503T	0	DaMaging
Chr2	MKI67IP	Missense	V228M	0.03	DaMaging
Chr7	MLL3	Missense	C988F	0	DaMaging
Chr11	MUC6	Missense	T1911M	0.04	DaMaging
Chr4	NHEDC1	Missense	A473V	0.01	DaMaging
Chr4	UGT2B11	Missense	H481R	0.02	DaMaging
Chr19	ZNF285	Missense	P455Q	0.03	DaMaging
Chr12	ZNF705A	Missense	R67W	0.04	DaMaging
Chr11	ZNHIT2	Missense	R384W	0.02	DaMaging

**Table 3:** Three out of 13 identified gene mutations (CABP4, FAM72D, MK167IP) in the ADNFLE pedigree were confirmed by Sanger sequencing

Individual	*CaBP4* (G155D)	*FAM72D* (G82V)	*MKI67IP* (V228M)
II4	+	+	+
III1	+	-	-
III3	+	+	+
III5	+	+	+
III7	+	+	+
III9	-	+	+
IV1	+	+	+
IV2	-	+	-
IV3	-	-	+
IV4	-	+	+
IV5	+	+	+
IV6	+	+	+
IV7	-	+	+
IV9	-	+	+

**Figure 1:**
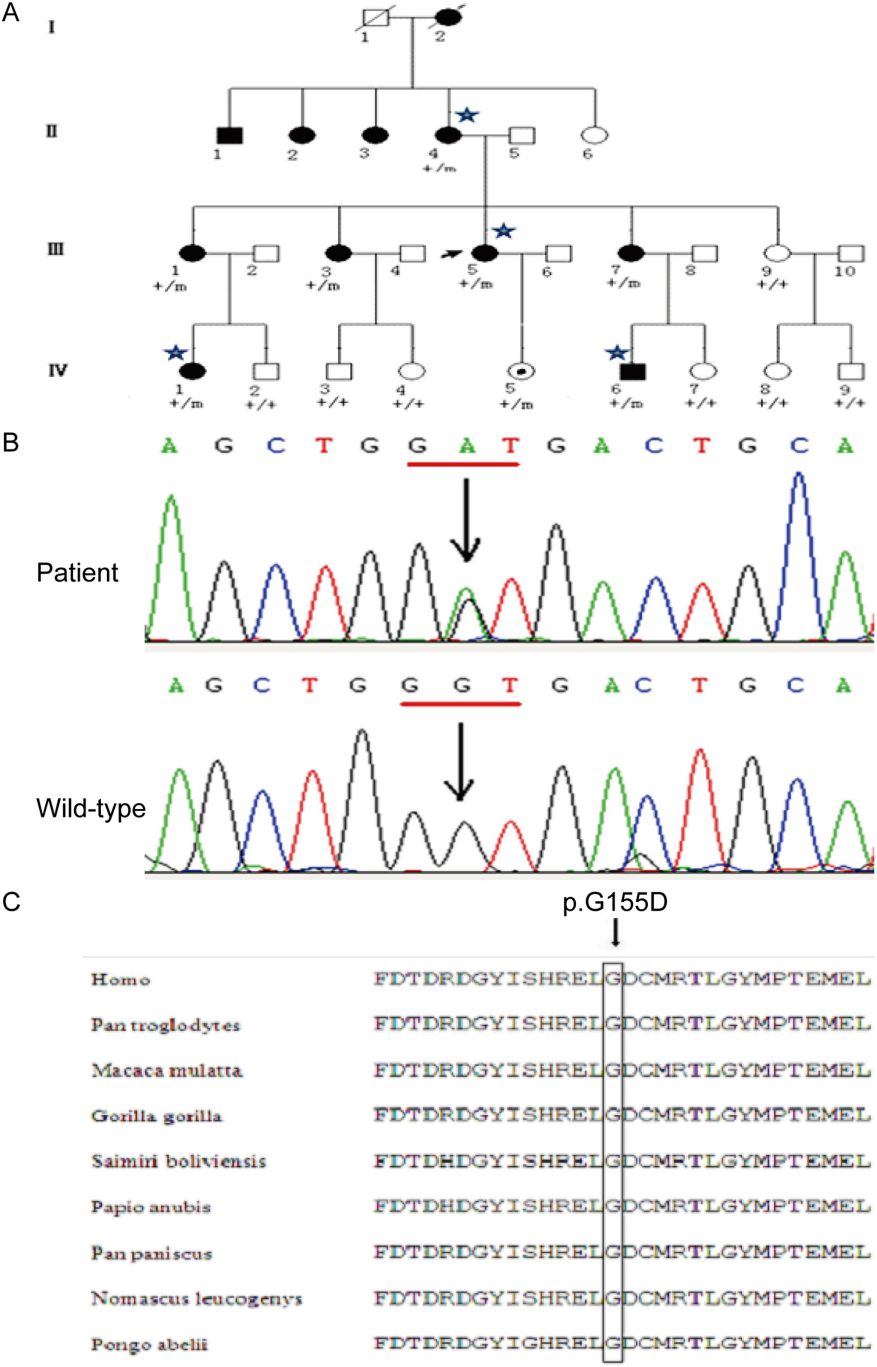
A Chinese ADNFLE pedigre with a mutation in the *CABP4* gene **(A)** ADNFLE pedigree with c.464G>A mutation in the CABP4 gene. Filled symbols indicate affected individuals and clear symbols unaffected individuals; squares: males; circles: females; arrow represents the proband, and asterisks mark the individuals for whom whole-exome sequencing was performed. Genotypes are indicated for each individual. “**+**” means wild type; “m” means mutant. **(B)** The c.464G>A mutation (↓) replaces D (glycine) with G (aspartic acid). **(C)** Evolutionary conservation of glycine G155. Black arrows indicate the positions of the missense mutations.

Finally, only one mutation was present in all seven affected members in this family: the c.464G>A (p.G155D) mutation in the Ca^2+^*-binding protein* 4 (*CABP4*) gene (OMIM: 608965) (Figure 1). Although, the mutation existed in an unaffected, one year old individual, the member of the pedigree was too young to meet the average age of onset of ADNFLE. The c.464G>A mutant in the *CABP4* gene was absent from the other unaffected individuals of the pedigree, and from 300 control cases.

**Figure 2:**
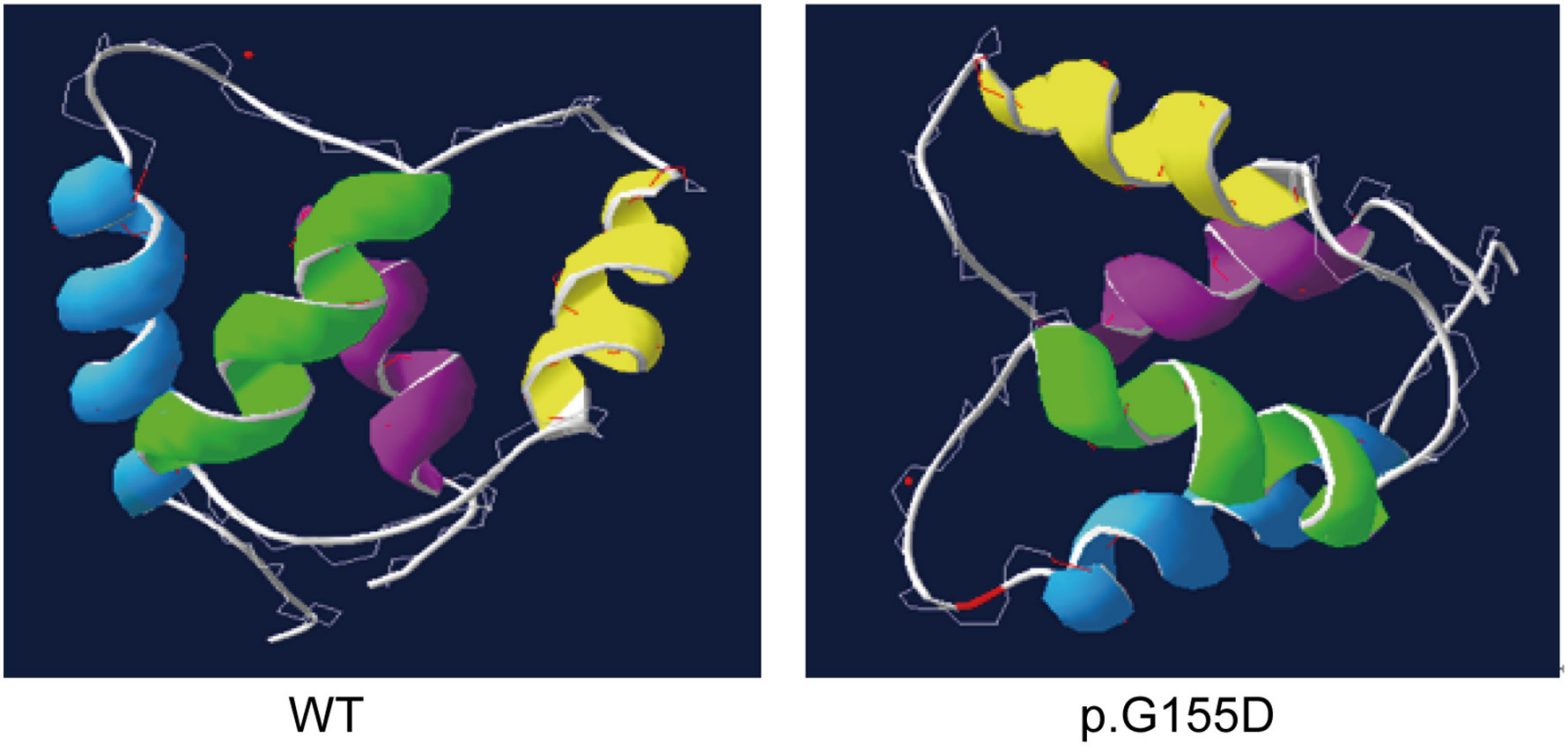
The three dimensional model of the CaBP4 protein containing the four EF-hand motifs (EF1-blue, EF2-green, EF3-yellow, and EF4-cyan) as revealed by the automated homology modeling program ESyPred3D Left: wild-type (WT) protein; right: p.G155D mutant protein.

The c. 464G>A substitution in the *CABP4* gene replaces a glycine residue at position 155 with an aspartic acid residue (p.G155D). This missense mutation is predicted to affect the CABP4 protein function according to evaluation with the bioinformatics tools PolyPhen 2 and SIFT (Prediction score 0.00). Furthermore, the wild-type glycine residue which is affected by the p.G155D mutation is highly conserved between primate mammal species (Figure 1C). The three dimensional structure of the CaBP4 protein with the novel missense mutation was modeled using the ESyPred3D web server (Figure 2). The c.464G>A substitution replaces a glycine residue with an aspartic acid residue, additionally containing an acidic side chain, at position 155, which is within the first Ca^2+^ binding EF-hand motif (Figure 2).

## DISCUSSION

In the present study, a novel missense mutation c.464G>A (p.G155D) was identified in the neuronal Ca^2+^ binding protein *CABP4* using whole-exome sequencing, and was found in all seven affected individuals from a 4-generation ADNFLE pedigree. This mutation was also found in one young, pre-symptomatic family member, but was absent from 300 unrelated control subjects. The mutation was localized in the EF hand Ca^2+^ binding domain and predicted to affect CABP4 protein function according to the bioinformatics tools PolyPhen 2 and SIFT.

CaBP4, which consists of 275 amino acids, is encoded by six exons on chromosome 11q11.3. CaBP4 belongs to the family of neuronal Ca^2+^-binding proteins (CaBPs) and shares structural homology with calmodulin. It has been shown to modulate voltage-dependent Ca^2+^ channels [9, 10]. CaBP4 contains four EF-hand Ca^2+^-binding motifs, but the lysine residue at position 1 of EF-hand 2 is not suitable for Ca^2+^ coordination; thus, the second EF-hand motif cannot coordinate Ca^2+^ [11].

**Figure 3:**
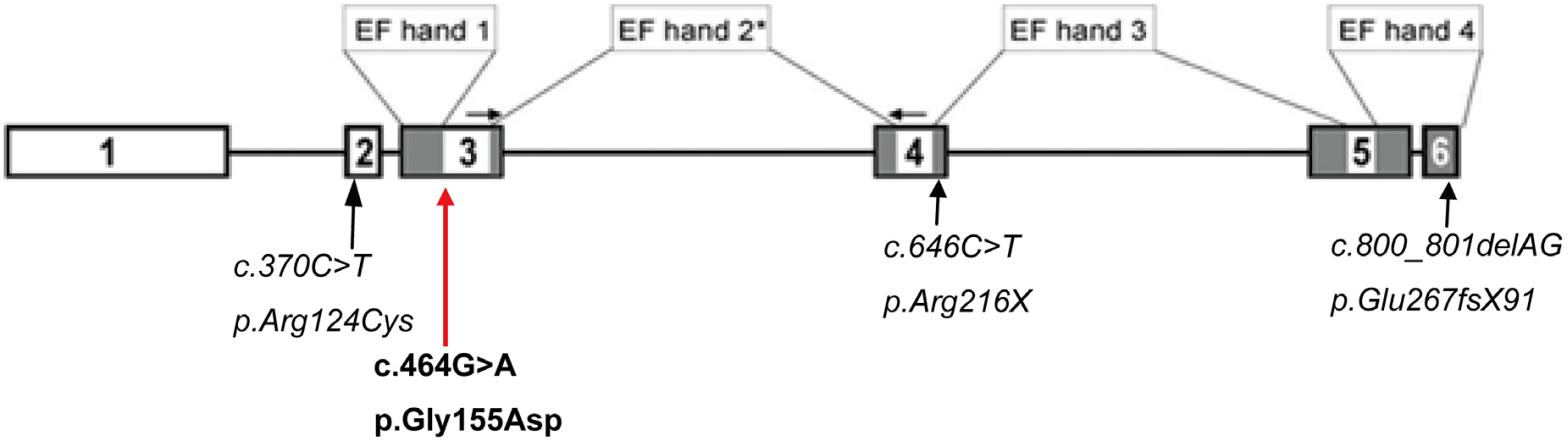
Schematic drawing of the *CABP4* gene Boxes: exons; lines: introns; gray boxes: parts of the gene that encode the EF hands EF1-4, the Ca^2+^-binding elements in CaBP4; In bold is the c.464G>A mutation and in italic are the previously identified mutations in CABP4. *EF hand 2 is not functional [10].

To date, three mutations have been identified in CaBP4 (Figure 3). All of them were detected in patients with retinal diseases, such as congenital stationary night blindness [12, 13, 14]. CaBP4 co-localizes with the voltage-gated L-type Ca^2+^ channel Cav1.4 in retinal synaptic terminals and regulates Ca^2+^ influx through the Ca^2+^ channel by binding to the ICDI (inhibitor of Ca^2+^-dependent inactivation) of CaV1.4. Thereby CaBP4 inhibits Cav1.4 inactivation and increases Ca^2+^ concentrations in synapses leading to enhanced neurotransmitter release [15]. Functional research of the CaBP4 mutations in congenital stationary night blindness have shown that the regulatory function of the mutant proteins is weaker compared to the wild type proteins [13, 16].

Interestingly, the N- and C-lobes of Ca^2+^-bound CaBP4 bind cooperatively to the IQ peptide of Cav1.4 [17]. The p.G155D mutation is located in the N-lobe of CaBP4 within EF1, which forms hydrophobic contacts and a salt bridge with the IQ domain of Cav1.4. Specifically, the CaBP4 residues Phe137 and Glu168 interact with Phe1586 and Arg1597, respectively, in Cav1.4.

Since the mutated p.G155D protein is predicted to bend and fold less easily if an aspartic acid exists at the G155 position, because of the additional acidic side chain, it is likely that the mutation induces structural changes in the Cav1.4 binding domain of CaBP4 between Phe137 and Glu168. These changes may interfere with cooperative binding to Cav1.4 or affect Ca^2+^ binding to the first EF-hand motif, which both may result in a weaker regulatory function. Therefore, it is tempting to speculate that the mutant protein decreases ion channel activation, leading to reduced Ca^2+^ concentrations, and thereby, for instance, interferes with the release of inhibitory neurotransmitters, thus, contributing to the etiology of ADNFLE function.

In summary, a novel missense mutation in *CaBP4* was identified in patients of a Chinese Han ADNFLE pedigree by means of whole-exome sequencing. The c.464G>A mutation is the first *CaBP4* mutation reported relating to ADNFLE, may provide a new insight into the pathology of ADNFLE. Further studies are necessary to identify CaBP4 mutations in other ADNFLE families, and functional investigations are necessary to predict the impact of the mutation in protein function and for the etiology of ADNFLE.

## MATERIALS AND METHODS

### Patients

In strict accordance with the classification criteria for the diagnosis of epilepsy and epilepsy syndrome by the 2010 International League Against Epilepsy (ILAE), we recruited 42 patients from 11 families with epilepsy and epilepsy syndrome. Diagnosis of these patients was based on the clinical history, EEG/ictal VEEG, video polysomnographic analysis, neurological examination and cranial magnetic resonance imaging (MRI). Among these families, a 4-generation pedigree from the Southern Chinese Han population, consisting of 27 members and 11 affected individuals, was diagnosed with ADNFLE (Figure 1). Fifteen family members participated in this study, including seven affected individuals and eight unaffected individuals. None of these patients had night blindness or visual disorders. The average age at onset was 11 years, ranging from 6 - 15 years. Three hundred healthy volunteers without epilepsy and related diseases were recruited as controls from the Southern Chinese Han population. Written informed consent was obtained from all study subjects and from healthy control individuals after explanation of the nature and possible consequences of the study. The study protocol was approved by the Ethics Committee for Human Research of the Guangdong General Hospital, and was in accordance with the tenets of the Declaration of Helsinki. Information was also obtained retrospectively from medical records.

### Exome sequencing and analysis

DNA was extracted from subjects’ peripheral blood using a QIAamp Blood DNA Kit (Sango Inc., Shanghai, China). Four affected individuals from the ADNFLE pedigree (II-4, III-5, IV-1 and IV-6, Figure 1) were selected for exome sequencing. Exome capture was performed with a SureSelect Human All Exon Kit. Exon-enriched DNA was sequenced on an Illumina Genome Analyzer II platform in accordance with the manufacturer’s instructions (BGI Inc., Shenzhen, China). The resulting sequences were further analyzed using the SOAP (Short OligonucleotideAnalysis Package) software, which allows the fast and accurate alignment for huge amounts of short reads generated by the Illumina Genome Analyzer (http://soap.genomics.org.cn/).

### Sequencing analyses and polymerase chain reaction (PCR)

To confirm the presence of gene variants identified via exome sequencing, and to screen for these variants in relatives and control subjects, genomic DNA derived from blood samples from 15 individuals from the ADNFLE pedigree and 300 control subjects was used for PCR amplification of the *CaBP4* gene comprising the c.464G>A mutant. PCR primers for the *CaBP4* gene comprising the c.464G>A mutant were ‘shown in Table 4) and were purchased from Sigma Inc, USA. PCR was performed using standard protocols in the 9700 PCR cycler (ABI Inc., USA). Sequencing of PCR amplification products was performed using an Applied Biosystems 3730XL DNA Analyzer (ABI Inc., Carlsbad, USA).

**Table 4:** Primers sequence and annealing temperature of exons of CABP4 gene

Exon	5’– 3’	3’– 5’	Temperature (°C)
1	GCAGAGGACTGGGATTAGGG	GGGAGGGTTATTGCTGCTC	20
1	ATGACCACAGAGCAGGCAAG	TCAGTCTGGGGCAGAAGAG	20
3	CGGGTGTTTCTTCCTAGGTG	CTGGACGGATCCTCTAGCTG	20
4	CAGCCTGGTGACAGAGCA	ACAAACTCTGCGGGAGAGAA	18
5	GAGTCTCCTTCCCGGAAAAC	CAGCCATCCTCTCCATTCAT	20

### Bioinformatics tools

PolyPhen-2 (Polymorphism Phenotyping, v2.2.2) is a bioinformatics tool that predicts possible impact of an amino acid substitution on the structure and function of a human protein using straightforward physical and comparative considerations [18]. The software uses protein sequences from the UniProtKB database (UniRef100), structures from PDB/DSSP Snapshot (78,304 entries), and UCSC MultiZ multiple alignments of 45 vertebrate genomes with the hg19/GRCh37 human genome (http://genetics.bwh.harvard.edu/pph2/). SIFT (Sorting Intolerant From Tolerant, v 5.2.2) predicts whether an amino acid substitution affects protein function based on sequence homology and the physical properties of amino acids [19]. Three dimensional model of CaBP4 was simulated with ESyPred3D (Lambert et al., 2002), a new automated homology modeling program (http://www.unamur.be/sciences/biologie/urbm/bioinfo/esypred/) [20].
